# NRT1.1-Mediated Nitrate Suppression of Root Coiling Relies on PIN2- and AUX1-Mediated Auxin Transport

**DOI:** 10.3389/fpls.2020.00671

**Published:** 2020-06-04

**Authors:** Sen Chai, En Li, Yan Zhang, Sha Li

**Affiliations:** State Key Laboratory of Crop Biology, College of Life Sciences, Shandong Agricultural University, Tai’an, China

**Keywords:** asymmetric root growth, nitrate, auxin transport, PIN2, AUX1, NRT1.1

## Abstract

Asymmetric root growth (ARG) on tilted plates, or root coiling on horizontally placed plates, is proposed to be a combination of gravitropism, mechanical sensing, and “circumnutation,” a word designated by Charles Darwin to describe the helical movement that all plant organs make around the growth direction. ARG is developmentally controlled in which microtubule-regulating proteins and the phytohormone auxin participates. Nutrient deficiency influences ARG. However, it is unclear which nutrients play key roles in regulating ARG, what endogenous components are involved in responding to nutrient deficiency for ARG, and how nutrient deficiency is translated into endogenous responses. We report here that nitrate deficiency resulted in a strong ARG in Arabidopsis. Nitrate deficiency caused root coiling on horizontal plates, which is inhibited by an auxin transport inhibitor, and by mutations in *PIN-FORMED2* (*PIN2*) and *AUXIN RESISTANT 1* (*AUX1*). We further show that suppression of ARG by nitrate is mediated by the nitrate transporter/sensor NRT1.1. In addition, PIN2- and AUX1-mediated auxin transports are epistatic to NRT1.1 in nitrate deficiency-induced ARG. This study reveals a signaling pathway in root growth by responding to exogenous nitrate and relaying it into altered auxin transport.

## Introduction

Asymmetric root growth (ARG) is waving and skewing on tilted plates or in extreme situation as right-handed coils on horizontal plates. Although under no gravity and mechanical stresses, Arabidopsis roots make large loops to the right-hand side (Piconese et al., 2003), it is generally believed that ARG is a combination of gravitropism, thigmotropism, and circumnutation (Migliaccio and Piconese, 2001; Migliaccio et al., 2009, 2013). ARG must be an evolutionary invention to increase plant fitness. Gravitropism is the process by which plant roots grow toward the center of the earth and thus favors deep rooting for more efficient nutrient absorption. Thigmotropism is the movement of roots away from obstacles and therefore allows plants to avoid harsh growth condition. Circumnutation is the helical movement that all plant organs produce around the growth direction (Migliaccio and Piconese, 2001; Migliaccio et al., 2009, 2013).

ARG is a developmentally regulated process (Oliva and Dunand, 2007; Roy and Bassham, 2014). Using the model plant *Arabidopsis thaliana*, many genetic factors affecting ARG have been characterized. Skewing the growth of the roots is usually accompanied by twisty epidermal cell files in the opposite direction, in which cortical microtubule (MT) organization plays a key role (Buschmann et al., 2004; Ishida et al., 2007; Bisgrove et al., 2008). Indeed, many MT-related proteins are involved in ARG, such as MT plus end-binding proteins EB1 (Bisgrove et al., 2008) and SKU6/SPIRAL1 (Sedbrook et al., 2004), as well as variants of α-tubulin (Ishida et al., 2007). MT-related mutants are usually defective in gravitropism or thigmotropism (Ishida et al., 2007; Bisgrove et al., 2008). By contrast, a few other genetic factors whose mutations resulted in enhanced ARG do not participate in the responses to gravity or mechanical stresses. For example, two seven-transmembrane proteins, the Mildew Resistance Locus O (MLO) family members, have been demonstrated to mediate ARG (Chen et al., 2009; Bidzinski et al., 2014). The heterotrimeric G proteins XLG3 and AGB1 positively regulate root waving and skewing while maintain normal responses to gravity and touch (Pandey et al., 2008), suggesting that they are specifically involved in the circumnutating behavior of roots.

Extensive studies have also demonstrated that ARG requires polar auxin transport (Piconese et al., 2003; Oliva and Dunand, 2007; Pandey et al., 2008; Chen et al., 2009; Qi and Zheng, 2013; Bidzinski et al., 2014). The phytohormone auxin plays an instructive role in establishing and maintaining plant cell and tissue polarity (Benkova et al., 2003; Boutte et al., 2007). During gravitropism, root curvature involves coordinated and asymmetric cell elongation between the lower and upper sides of the roots (Abas et al., 2006; Baster et al., 2013). This process is mediated by differential cellular auxin levels that are generated by activities of the auxin carriers, such as PIN-FORMED2 (PIN2) and AUXIN RESISTANT 1 (AUX1) (Chen et al., 1998; Luschnig et al., 1998; Marchant et al., 1999; Swarup et al., 2001, 2005; Blilou et al., 2005; Abas et al., 2006). PIN2 and AUX1 are required for polar auxin to transport away from the root tip into more differentiated tissues (basipetal transport) during root gravitropism, while AUX1 specifically contributes to polar auxin transport in the lateral root cap and epidermal layers (Swarup et al., 2001, 2005; Boutte et al., 2007). Polar auxin transport may regulate cell wall dynamics and intracellular trafficking, thus promoting ARG (Roy and Bassham, 2014).

ARG is also controlled by environmental cues, especially nutrients (Buer et al., 2000, 2003; Oliva and Dunand, 2007). Zero salt induces the most wavy growth of Arabidopsis roots on 45 degree–tilted plates (Buer et al., 2000), suggesting that some nutrients in the growth medium suppress the circumnutating behavior of roots. However, it is unclear what nutrients play key roles in suppressing ARG (root waving and skewing, and in extreme cases root coiling and spiralization). Second, it is unclear what endogenous components are involved in responding to nutrient deficiency for ARG. Finally, how the perception of nutrient deficiency is translated into endogenous responses is also obscure.

We report here that nitrate deficiency resulted in a strong ARG in Arabidopsis. Nitrate deficiency caused root coiling on horizontal plates, which is inhibited by the auxin transport inhibitor 1-*N*-naphthylphthalamic acid (NPA), and by loss-of-function mutations in *PIN2* or *AUX1*. We further show that the suppression of ARG by nitrate is mediated by the nitrate transporter and sensor NITRATE TRANSPORTER 1.1 (NRT1.1). Nitrate deficiency and the functional loss of *NRT1.1* both resulted in asymmetric distribution of PIN2 and enhanced asymmetric auxin signaling, leading to root coiling. This study reveals a signaling pathway in root growth by responding to nitrate deficiency and relaying it into intracellular activities through altered auxin transport.

## Materials and Methods

### Plant Materials and Growth Conditions

Arabidopsis Columbia-0 ecotype was used as the wild type. Mutants and transgenic lines including *pin2*/*eir1-1* (Chen et al., 1998; Luschnig et al., 1998), *pin3-5* (Blilou et al., 2005), *pin7-2* (Blilou et al., 2005), *aux1-7* (Swarup et al., 2004), *nrt1.1/chl1-5* (Huang et al., 1999), *chl1-13* (Wang et al., 2009), *chl1-9* (Ho et al., 2009), *nrt1.2* (Huang et al., 1999), *nrt2.1-2* (Wang and Crawford, 1996), AUX1:YFP (Swarup et al., 2005), *Pro_*CycB*__1__;__1_*:GUS (Colon-Carmona et al., 1999), *Pro_35__*S*_*:GFP-MAP4BD (Marc et al., 1998), DR5:GFP (Benkova et al., 2003), and PIN2:GFP (Xu and Scheres, 2005) were described previously. For seedlings growing on plates, Arabidopsis seeds were surface-sterilized and plated on 1/2 Murashige and Skoog basal medium (MS) supplemented with vitamins (Phytotechlab^1^) except where noted.

### Quantitative Analysis of ARG

Arabidopsis seeds were first vernalized on 1/2 MS medium for 4 days and then transferred to MRGL medium (Fukuda et al., 2016) without potassium (K^+^ replaced by Na^+^), calcium (Ca^2+^ replaced by Na^+^), sucrose (0% instead of 1%), or nitrate (NO_3_^–^ replaced by Cl^–^). Phosphorus-deficient medium was prepared by replacing KH_2_PO_4_ with KCl from a medium (5 mM KNO_3_, 20 mM NH_4_NO_3_, 2 mM MgSO_4_, 1 mM CaCl_2_, 0.1 mM Fe-EDTA, 50 μM H_3_BO_4_, 12 μM MnSO_4_, 1 μM ZnCl_2_, 1 μM CuSO_4_, 1 mM KH_2_PO_4_ (pH 5.5), 0.2 μM Na_2_MoO_4_, and 1% (w/v) sucrose). After being vertically placed for 2 days, the plates were placed horizontally in the dark for 5 days before analyses of the growth axis or kept vertically for 5 days before the measurement of the primary root growth. The growth axis of the primary roots immediately after being switched from the vertical position to the horizontal position was set as the starting angle. The growth axis of the primary roots after being horizontally placed for 5 days was set as the end angle. The degree of ARG was defined as the total angle the root apex has turned during the 5 days (for example, one complete coil indicates 360 degrees). For all experiments measuring ARG, three independent experiments involving 30 roots in each genetic background or treatment were conducted. Quantification of the angles was performed using ImageJ^2^. Statistical analyses were performed with GraphPad Prism 6^3^ with build-in tools and parameters.

### RT-PCRs and Quantitative Real-Time PCRs

The total RNAs of the roots from the 4 days-after-germination (DAG) seedlings were isolated using a Qiagen RNeasy plant miniprep kit according to the manufacturer’s instructions. Oligo(dT)-primed cDNAs were synthesized using Superscript III reverse transcriptase with on-column DNase digestion (Invitrogen). Quantitative real-time PCRs were performed with the Bio-Rad CFX96 real-time system using a SYBR Green real-time PCR master mix (Toyobo) as described (Zhou et al., 2013). All the primers are listed in Supplementary Table S1.

### GUS Histochemistry

Seedlings were incubated for 4 h with 5-bromo-4-chloro-3-indolyl-D-GlcUA (X-Gluc) in the dark and examined with an Olympus BX53 microscope as described (Zhou et al., 2013).

### Pharmacological Treatments

For the effects of NPA on root growth, seedlings at 4 DAG were transferred from regular 1/2 MS plates to MGRL or MGRL without nitrate supplemented with NPA at a final concentration of 10 μM. DMSO was similarly diluted as the control. After incubation, roots were examined with an Olympus BZX16 microscope.

### Fluorescent Microscopy and Quantification

For CLSM imaging of GFP-, YFP-fusions, and FM4-64, excitation/emission is 488 nm/505–550 nm, 515 nm/530–580 nm, and 561 nm/600–650 nm, respectively, using the super-resolution confocal microscopy LSM880 (Zeiss). Image captures were performed with the same confocal settings (gain, laser strength, pinhole) to generate comparable images among different treatments or genetic backgrounds. Images were exported and processed using Adobe Photoshop CS3 (Adobe). ImageJ (see text footnote 2) was used to quantify the intensities of fluorescence signals at the PM or in BFA compartments.

### Accession Numbers

Arabidopsis Genome Initiative locus identifiers for the genes mentioned in this article are: *AUX1*, AT2G38120; *BRI1*, AT4G39400; *NIA1*, AT1G77760; *NRT1.1*, AT1G12110; *NRT1.2*, AT1G69850; *NRT2.1*, AT1G08090; *PIN2*, At5G57090; *PIN3*, AT1G70940; *PIN7*, AT1G23080.

## Results

### Nitrate Deficiency Induces Root Coiling on Horizontal Plates

To determine which nutrients within the growth media suppress ARG, we tested the effect of various media compositions, each time leaving out different essential nutrients. MRGL medium was modified by replacing K^+^ with Na^+^ (-K^+^), replacing Ca^2+^ with Na^+^ (-Ca^2+^), replacing NO_3_^–^ with Cl^–^ (-NO_3_^–^), or by excluding sucrose (-Suc) to test the effect of potassium, calcium, nitrate, and sucrose deficiency on ARG. To simplify visualization and quantification, plates were placed horizontally because it allowed the growth of roots into coils (Chen et al., 2009; Bidzinski et al., 2014).

**FIGURE 1:**
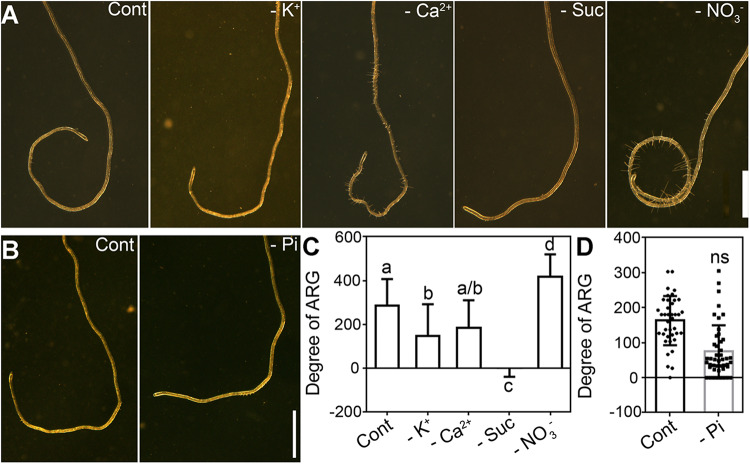
Nitrate deficiency enhances asymmetric root growth (ARG) on horizontal plates. **(A)** A representative primary root of Arabidopsis growing on MGRL medium (Cont), or MGRL medium deficient in potassium (-K^+^), calcium (-Ca^2+^), sucrose (-Suc), or nitrate (-NO_3_^–^). **(B)** A representative primary root of Arabidopsis growing on 1 mM phosphorus medium (Cont) or phosphorus-deficient medium (-Pi). **(C,D)** Degree of ARG. The y-axis indicates coiling degree, i.e., 360 degree for one complete coil. Right-hand turning is defined as positive values while left-hand turning as negative values. Results are means ± standard error (SE). Three independent experiments involving 30 roots in each treatment were conducted. Means with different letters are significantly different (One-way ANOVA, Tukey’s multiple comparisons test, *P* < 0.05) for **(C)**. ns, not significantly different. Bars = 1 mm for **(A,B)**.

On control plates containing MGRL medium (Figure 1A) or 1 mM phosphorus-medium (Figure 1B), roots made an average of 300-degree turn. Deficiency of potassium, calcium, sucrose, or phosphorus did not significantly enhance such ARG (Figures 1A–D). By contrast, nitrate deficiency resulted in a strong root coiling phenotype (Figure 1A), significantly differing from that of control medium (Figure 1C). Because ARG can be influenced by root growth, by responses to gravity or mechanical stresses, especially on horizontal plates, we performed several experiments to determine whether these factors also contributed to the strong ARG induced by nitrate deficiency. First, quantitative analysis showed that nitrate deficiency did not affect primary root growth (Supplementary Figure S1). Rather, deficiency in potassium, sucrose, or phosphorus significantly reduced primary root growth (Supplementary Figure S1), which might have caused a reduced ARG (Figures 1A–D). Second, gravitropism was slightly affected at 6 h upon gravity stimulation by nitrate deficiency (Supplementary Figure S2). However, no significant difference was detected after 6 h, i.e., the final turning angles were comparable between roots growing on MGRL medium (thereafter as 7N) and MGRL -NO_3_^–^ medium (thereafter as 0N) (Supplementary Figure S2). Third, we tested the effect of mechanical stress on nitrate deficiency-induced ARG by adjusting the tilting angles of agar plates according to previous reports (Buer et al., 2000; Shih et al., 2014). The increase of titling angles enhanced root skewing both on 7N and 0N plates (Supplementary Figure S3), excluding the contribution of mechanical stimulation in nitrate deficiency-induced ARG. These results suggested that the slightly affected gravitropism and thigmotropism were not the major cause of the significantly different ARG by nitrate deficiency.

Because nitrate is one of the nitrogen sources plants utilize (Kiba et al., 2011), we wondered whether it was the general reduction of available nitrogen sources or nitrate specifically that induced the stronger ARG. Therefore, we supplied 100 mM L-glutamate (Glu) or 5 mM NH_4_^+^ in 0N medium to determine whether other nitrogen sources were able to suppress the strong ARG. Addition of Glu or NH_4_^+^ did not suppress the stronger ARG on 0N medium (Supplementary Figure S4). Rather, addition of Glu or NH_4_^+^ slightly enhanced ARG on 0N medium (Supplementary Figure S4). The results suggested that the other nitrogen sources could not replace nitrate in suppressing ARG.

### Nitrate Deficiency-Induced Root Coiling Results From Unequal Cell Division and Elongation

**FIGURE 2:**
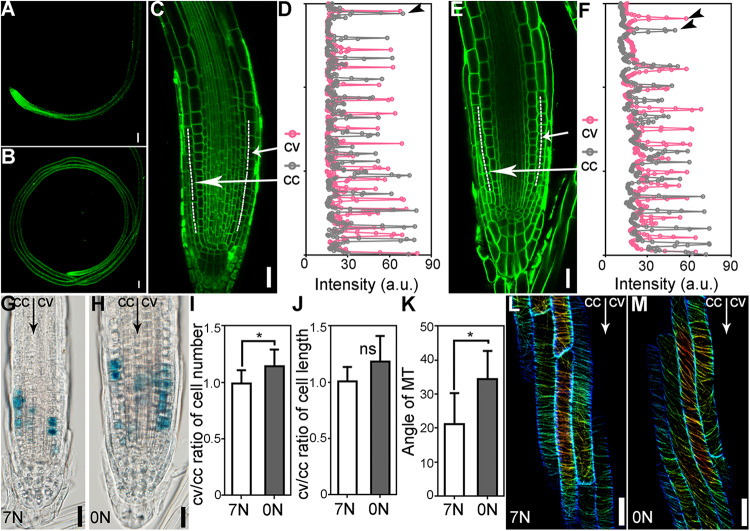
Nitrate deficiency-induced ARG results from unequal cell division and elongation. **(A,B)** A primary root on 7N **(A)** or 0N medium **(B)**. FM4-64 staining (green) was used to show cell silhouettes. **(C–F)** Representative root meristem zone on 7N **(C,D)** or 0N **(E,F)**. Confocal laser scanning micrographs (CLSM) are shown in **(C,E)**. Signal intensity of the dotted lines across 14 epidermal cells either from the convex side (cv) or the concave side (cc) in **(C,E)** is shown in **(D,F)**, respectively. Peaks in **(D,F)** indicate the plasma membrane (PM) of cells. The distance between two neighboring peaks of the same color indicates the length of a cell. **(G,H)** Representative histochemical GUS staining of *Pro*_*CYCB*_:GUS roots growing on 7N **(G)** or on 0N medium **(H)**. All 15 roots examined for each treatment (7N or 0N) showed similar results. **(I,J)** Ratio of cv/cc cell numbers **(I)** or length **(J)**. Only cells at the elongation zone were measured. **(K)** Angle of MT to the transverse plane of root growth axis. Results shown in **(I–K)** are means ± SE. Three independent experiments involving 30 roots in each treatment were conducted. Asterisks indicate significant difference (*t*-test, *P* < 0.05). ns indicates no significant difference (*t*-test, *P* > 0.05). **(L,M)** CLSM of a primary root from the *Pro_35__*S*_*:GFP-MAP4BD transgenic plants on 7N **(L)** or 0N medium **(M)** 8 h after being placed horizontally. Bars = 100 μm for **(A,B)**, 20 μm for **(C,E,G,H,L,M)**.

Both unequal cell division and elongation could result in ARG. To determine which contributed to ARG upon nitrate deficiency, we pulse-labeled Arabidopsis roots that growing on the 7N or 0N horizontal plates for 5 days with the lipophilic dye FM4–64 (Figures 2A–C,E), which labels the PM initially. Arabidopsis roots made an average of 300-degree turn on the 7N plates (Figure 2A). By contrast, they formed closed coils on the 0N plates (Figure 2B). Although average cell length of root epidermal cells showed no significant difference between the convex and concave sides (Figure 2J), close-examination of root epidermal cells at the elongation zone indicated that roots growing on 0N plates contained a higher number of large cells at the convex side than at the concave side (Figures 2D,F,I), indicating unequal cell elongation.

On the other hand, histochemical GUS staining of the *Pro_*C*__*yc*__*B*__1__;__1_*:GUS seedlings showed that even before the right-hand turning of root tips, there was already more cell division at the R side compared to the L side along the growth axis (Figures 2G,H). Quantification of epidermal cell numbers within root meristematic zone along either the convex side or the concave side showed a slight but significant increase at the convex side of roots growing on 0N (Figure 2I). These results indicated that ARG upon nitrate deficiency associates with unequal cell division.

Because dynamic MT deposition was usually associated with ARG (Buschmann et al., 2004; Ishida et al., 2007; Bisgrove et al., 2008), we examined MT dynamics upon nitrate deficiency by analyzing the distribution of GFP-MAP4BD, a fluorescence probe for MT in plant cells (Marc et al., 1998). In elongating epidermal cells of the roots growing on 7N medium, MT showed a pattern nicely transverse to the growth direction (Figures 2K,L). By contrast, MT was more skewed in epidermal cells of roots growing on 0N medium (Figures 2K,M), consistent with the critical role of MT organization in maintaining the direction of epidermal cell files (Sedbrook et al., 2004). To provide further support to the involvement of MT during root coiling, we took a pharmacological approach by investigating the effect of MT-depolymerizing or stabilizing drug, oryzalin, or taxol on root coiling. Either oryzalin or taxol suppressed nitrate deficiency-induced root coiling (Supplementary Figure S5), suggesting an important role of dynamic MT organization in nitrate deficiency-induced ARG.

### Nitrate Deficiency-Induced ARG Requires Polar Auxin Transport

**FIGURE 3:**
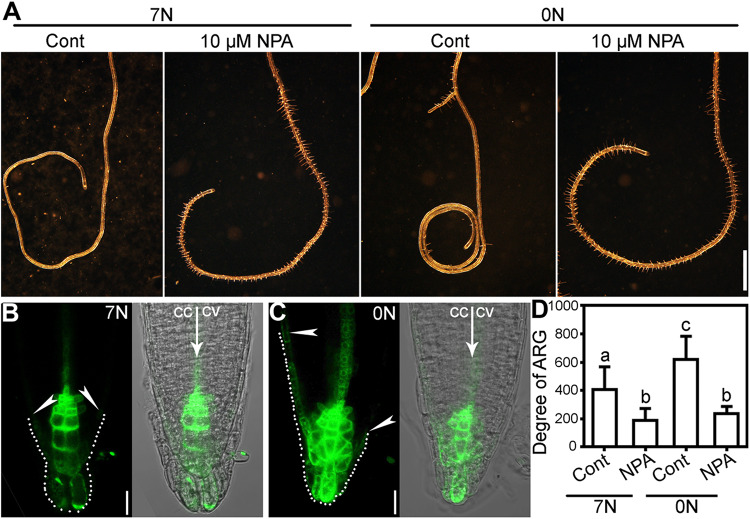
Nitrate deficiency-induced ARG depends polar auxin transport. **(A)** A representative root with NPA treatment (10 μM NPA) or without (cont) on 7N or 0N medium. **(B,C)** CLSM of DR5:GFP transgenic roots on 7N **(B)** or 0N **(C)** medium horizontally placed for 8 h. Merges of the GFP channel and the transmission channel are shown at the left side of each corresponding GFP image. cc, concave; cv, convex. All 15 roots examined for each treatment (7N or 0N) showed similar results. **(D)** Degree of ARG upon NPA treatment or mock treatment on 7N or 0N medium. Results are means ± SE. Three independent experiments involving 30 roots in each treatment were conducted. Means with different letters are significantly different (One-way ANOVA, Tukey’s multiple comparisons test, *P* < 0.05). Bars = 1 mm for **(A)**, 10 μm for **(B,C)**.

Because polar auxin transport was implicated in ARG induced by many genetic factors (Pandey et al., 2008; Chen et al., 2009; Qi and Zheng, 2013), we wondered whether it was also the situation for nitrate deficiency-induced ARG. To test this possibility, we first apply NPA, an inhibitor of polar auxin transport. NPA at 10 μM significantly suppressed root coiling under nitrate deficiency (Figures 3A,D), suggesting that polar auxin transport was required for the nitrate deficiency-induced ARG.

To determine whether nitrate deficiency affected auxin responses at the root tip, we analyzed the distribution of GFP signals using an auxin reporter line, DR5:GFP. Fluorescence distribution and intensity of the DR5:GFP transgenic plants have been widely adopted as the indicator of auxin responses (Ulmasov et al., 1997; Sabatini et al., 1999). We transferred seedlings of 4 days after germination (DAG) from regular vertical 1/2 MS plates to either horizontally placed 7N or 0N medium. CLSM was performed at 8 h after the transfer. On 7N medium, there was a symmetric distribution of GFP signals at the root tip (Figure 3B). By contrast, GFP signals were extended more basipetally at the left side, i.e., the future concave side, on 0N medium (Figure 3C), suggesting asymmetric auxin response induced by nitrate deficiency before ARG.

### Nitrate Deficiency-Induced ARG Depends on *PIN2* and *AUX1*

**FIGURE 4:**
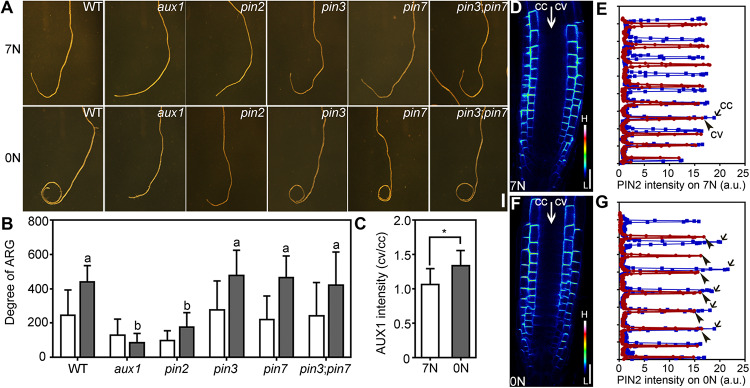
Nitrate deficiency-induced ARG depends on *PIN2* and *AUX1*. **(A)** Representative roots from wild type (WT), *aux1*, *pin2*, *pin3*, *pin7*, and *pin3;pin7* on 7N or 0N medium. **(B)** Degree of ARG on 7N (open bars) or 0N medium (filled bars). Values are means ± SE. Three independent experiments involving 30 roots in each genetic background were conducted. Different letters represent significantly different groups (One-Way ANOVA, Tukey’s multiple comparisons test, P < 0.05). **(C)** The intensity ratio of AUX1:YFP at convex vs. concave of the LRC on 7N or 0N medium. Asterisk indicates significant difference (*t*-test, *P* < 0.05). Values in **(B,C)** are means ± SE. Three independent experiments involving 30 roots in each genetic background were conducted. **(D–G)** CLSM **(D,F)** or fluorescence intensity **(E,G)** of epidermal cells of the PIN2:GFP root meristem zone on 7N **(D,E)** or 0N medium **(F,G)**. The GFP images are displayed as pseudocolors (H: high; L: low). In **(E,G)**, blue lines indicate the concave side (cc) while red lines indicate the convex side (cv). a.u. represents arbitrary fluorescence unit. Bars = 1 mm for **(A)**, 20 μm for **(D,F)**.

A major factor in asymmetric auxin distribution and responses is through the collaborative function of auxin efflux and influx carriers. Because NPA treatment abolished nitrate deficiency-induced ARG (Figure 3), we suspected that PIN2 and AUX1 were involved because they both participate in polar auxin transport (Boutte et al., 2007; Band et al., 2014). To test this hypothesis, we analyzed the degree of ARG of several auxin transport mutants upon nitrate deficiency, including *aux1*, *pin2/eir1-1*, *pin3*, *pin7*, and *pin3;pin7*, all of which have been demonstrated to mediate asymmetric growth (Chen et al., 1998; Luschnig et al., 1998; Friml et al., 2002; Swarup et al., 2005; Abas et al., 2006). Among all the mutants tested, *aux1* and *pin2* lost the ability to form root coils upon nitrate deficiency while *pin3*, *pin7*, and *pin3;pin7* behaved comparably with wild type (Figures 4A,B). All those auxin-related mutants showed comparable length of primary roots either on 7N medium or 0N medium (Supplementary Figure S6), excluding the influence of the primary root growth on ARG. This result suggested that ARG induced by nitrate deficiency requires *PIN2* and *AUX1*.

To determine how nitrate deficiency affected PIN2 and AUX1, we first tested their transcript abundance in 4 DAG seedlings growing on 7N medium or 0N medium. As a control for nitrate deficiency, the expression of *NITRATE REDUCTASE 1* (*NIR1*) was reduced on 0N medium (Supplementary Figure S7), as expected (Wang et al., 2009). There was a significant reduction of *PIN2* but not *AUX1* in nitrate-deficient seedlings compared to seedlings growing on 7N medium (Supplementary Figure S7). Next, we examined the distribution of AUX1 and PIN2 by analyzing the fluorescence intensity of AUX1:YFP and PIN2:GFP transgenic roots. A slight but significant increase of AUX1:YFP levels at the convex side of the lateral root cap (LRC) was observed after the roots were transferred to 0N medium horizontally (Figure 4C). PIN2 was asymmetrically localized at the PM of the root epidermal and cortex cells either on 7N or on 0N medium (Figures 4D,F). After being transferred to the 0N medium horizontally, differential PIN2:GFP intensity was detected at the two sides of the growth axis, i.e., signals were stronger at the concave side than at the convex side (Figure 4G). Such asymmetry was not detected on 7N medium (Figure 4E). These results suggested that nitrate deficiency affected the distribution of auxin transporters in roots.

### NRT1.1 Is Critical for Nitrate Suppression of ARG

Because nitrate suppresses the ARG, we wanted to determine what components translate exogenous nitrate into intracellular changes leading to the suppressed ARG. Extensive studies have demonstrated that several nitrate transporters, specifically NRT1.1, NRT1.2, and NRT2.1, participate in the root uptake of NO_3_^–^ (Kiba et al., 2012; Wang et al., 2012). In addition, NRT1.1 and NRT2.1 also act as nitrate sensors (Little et al., 2005; Ho et al., 2009; Gojon et al., 2011). To determine whether these key nitrate transporters or sensors were involved in the nitrate suppression of ARG, we compared the degree of ARG of wild type (WT) and the mutants of these transporters, i.e., *chl1-5* and *chl1-13*, *nrt1.2*, and *nrt2.1*, as the null mutants of *NRT1.1* (Liu et al., 1999), *NRT1.2* (Huang et al., 1999), and *NRT2.1* (Little et al., 2005), respectively. We also included *chl1-9* into the study, a mutant of *NRT1.1* in which NRT1.1 lost its nitrate uptake ability but retained its nitrate sensor function (Ho et al., 2009).

**FIGURE 5:**
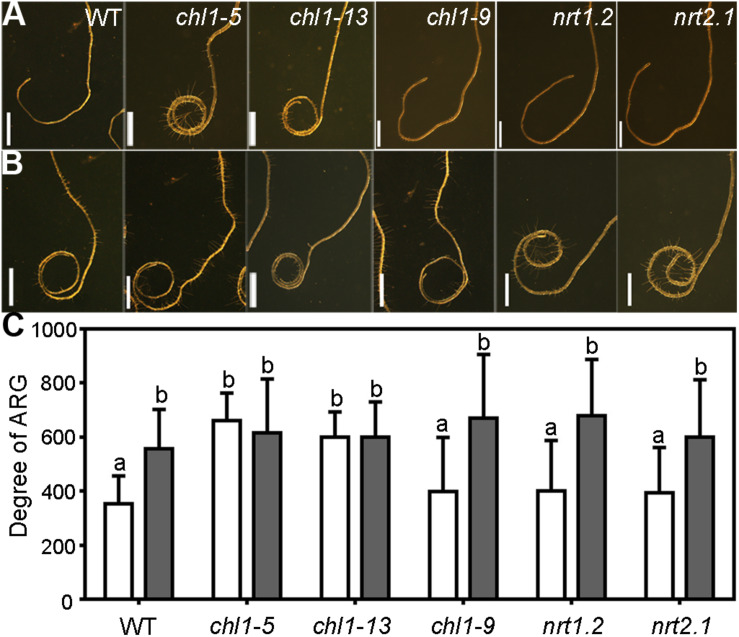
NRT1.1 is critical for nitrate suppression of ARG through its nitrate uptake function. **(A,B)** Representative roots from wild type (WT), *chl1-5*, *chl1-13*, *chl1-9*, *nrt1.2*, and *nrt2.1* on 7N medium **(A)** or 0N medium **(B)**. **(C)** Degree of ARG on 7N (open bars) or 0N medium (filled bars). Results are means ± SE. Three independent experiments involving 30 roots in each treatment were conducted. Means with different letters are significantly different (One-way ANOVA, Tukey’s multiple comparisons test, *P* < 0.05). Bars = 1 mm.

On the 7N medium, wild-type roots made an average of 300-degree right turn after 5 days on horizontal plates, which was significantly enhanced on 0N medium (Figures 5A,B), as shown before (Figure 1). Both *nrt1.2* and *nrt2.1* behaved just like wild type such that an enhanced ARG was observed on 0N medium as compared to that on the 7N medium (Figures 5A,B), suggesting that both nitrate transporters were not essential for the nitrate suppression of ARG. The mutants *chl1-5* and *chl1-13* formed closed coils even on the 7N medium (Figure 5A) and showed comparable ARG no matter whether nitrate was supplied (Figures 5A,B). By contrast, *chl1-9*, in which NRT1.1 lost its nitrate uptake function but not nitrate sensing role (Ho et al., 2009), was comparable to wild type (Figures 5A,B). Because nitrate transporter mutants did not significantly differ from wild type either in the length (Supplementary Figure S8) or the growth morphology of the primary roots (Little et al., 2005; Krouk et al., 2010) on vertical plates supplemented with sufficient nitrates, the influence of primary root growth on ARG in *NRT1.1* loss-of-function was excluded. The results suggested that NRT1.1 mediates the nitrate suppression of ARG, and its role in this process is likely through being the nitrate sensor.

We performed several experiments to determine whether responses to gravity or mechanical stress of *chl1-5* contributed to the strong ARG on the 7N medium. First, gravitropism was slightly affected in *chl1-5* at 6 h upon gravity stimulation (Supplementary Figure S9). However, no significant difference was detected after 6 h, i.e., the final turning angles were comparable between wild type and *chl1-5* on the 7N medium (Supplementary Figure S9). Second, *chl1-5* did not show different responses to mechanical stresses either on the 7N medium or the 0N medium (Supplementary Figure S10). These results suggested that the nitrate transporter/sensor NRT1.1 is essential for nitrate suppression of ARG not through gravitropism or thigmotropism.

### NRT1.1-Mediated Suppression of ARG Requires PIN2- and AUX1-Controlled Auxin Transport

**FIGURE 6:**
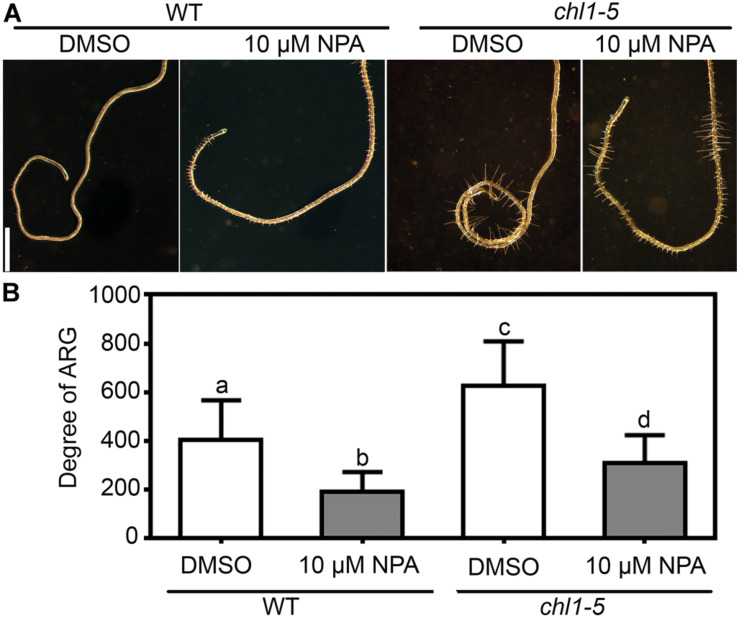
Enhanced ARG of *chl1-5* on nitrate-sufficient medium is suppressed by NPA. **(A)** Representative wild-type or *chl1-5* roots with NPA treatment (10 μM NPA) or without (cont) on 7N medium. Bars = 1 mm. **(B)** Degree of ARG upon NPA treatment or mock treatment on 7N medium. Results are means ± SE. Three independent experiments involving 30 roots in each treatment were conducted. Means with different letters are significantly different (One-way ANOVA, Tukey’s multiple comparisons test, *P* < 0.05).

**FIGURE 7:**
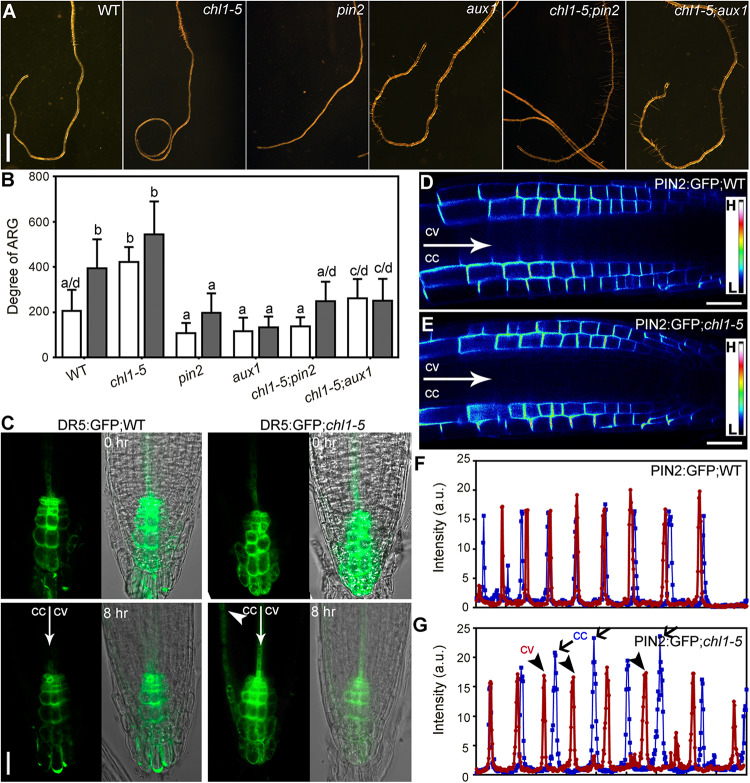
NRT1.1-mediated nitrate suppression of ARG requires PIN2- and AUX1-controlled auxin transport. **(A)** Representative roots from wild type (WT), *chl1-5*, *pin2*, *aux1*, *chl1-5;pin2*, and *chl1-5;aux1* on 7N medium. **(B)** Degree of ARG on 7N (open bars) or 0N medium (filled bars). Results are means ± SE. Three independent experiments involving 30 roots in each sample were conducted. Means with different letters are significantly different (One-way ANOVA, Tukey’s multiple comparisons test, *P* < 0.05). **(C)** CLSM of DR5:GFP transgenic roots on 7N medium horizontally placed for 0 or 8 h. Merges of the GFP channel and the transmission channel are shown at the left side. cc, future concave side; cv, future convex. The arrowhead points at where expanded GFP signals are. **(D,E)** CLSM of PIN2:GFP roots in wild type **(D)** or *chl1-5* **(E)** on 7N medium. The GFP images are displayed as pseudocolors (H: high; L: low). **(F,G)** Intensity of PIN2:GFP in wild type **(F)** or in *chl1-5* **(G)** along a comparable distance of root epidermal cells within the elongation zone on 7N medium. cc, concave side; cv, convex side. Peaks indicate the signals at the PM. Bars = 1 mm for **(A)**, 10 μm for **(C)**, 20 μm for **(D,E)**.

Because nitrate deficiency–induced ARG requires *PIN2* and *AUX1* (Figure 4), we wondered whether the NRT1.1-mediated nitrate suppression of the ARG also depended on the activity of *PIN2* and *AUX1*. To test this possibility, we first examined the effect of NPA on *chl1-5*. Application of NPA significantly suppressed the root coiling of *chl1-5* on the 7N medium (Figures 6A,B), suggesting that auxin transport was epistatic to nitrate sensing by NRT1.1. Next, we analyzed the genetic interaction between *chl1-5* and *pin2* or *aux1*. Indeed, either *pin2* or *aux1* suppressed the nitrate insensitive ARG of *chl1-5*, i.e., both the *chl1-5;pin2* and *chl1-5;aux1* double mutants failed to form root coils on the 7N medium, unlike the *chl1-5* single mutant (Figures 7A,B). These results suggested that ARG caused by functional loss of *NRT1.1* depends on *PIN2*- and *AUX1*-mediated polar auxin transport.

To determine whether functional loss of *NRT1.1* affected the functionality of *PIN2* and *AUX1*, we first tested their transcript abundance in roots of 4 DAG *chl1-5* seedlings growing on the 7N medium. Interestingly, the transcript abundance of *PIN2* was reduced in *chl1-5* on the 7N medium, comparable with that in wild type on the 0N medium (Supplementary Figure S7). Next, we examined the distribution of PIN2 by analyzing the fluorescence intensity of PIN2:GFP transgenic roots in wild type or in *chl1-5* on the 7N medium. PIN2 was asymmetrically localized at the PM of root epidermal and cortex cells both in wild type (Figure 7D) as in *chl1-5* (Figure 7E). After being placed horizontally, differential PIN2:GFP intensity was detected at the two sides of the growth axis, i.e., signals were stronger at the concave side than at the convex side on 7N medium in *chl1-5* (Figure 7G) but not in wild type (Figure 7F). Finally, we transferred 4 DAG seedlings of DR5:GFP or DR5:GFP;*chl1-5* from regular vertical MS plates to horizontally placed 7N medium. CLSM was performed at 8 h after transfer. There was a symmetric distribution of GFP signals at the root tip immediately after transfer both in wild type and in *chl1-5* root tips (Figure 7C). By contrast, GFP signals were more extended basipetally at the future concave side in *chl1-5* after 8 h when roots were yet to turn (Figure 7C), suggesting asymmetric auxin responses. There results suggested that NRT1.1-mediated nitrate suppression of ARG requires PIN2-mediated polar auxin transport.

## Discussion

We report here a genetic pathway controlling nutrient-mediated ARG in Arabidopsis. We show that nitrate deficiency (Figure 1) or functional loss of *NRT1.1* (Figure 5) enhances the circumnutation behavior of Arabidopsis roots such that it results in roots forming closed coils on horizontally placed plates rather than making wavy movement under nitrate-sufficient medium. Because root growth was evaluated under artificial situations, i.e., horizontal agar plates, whether such behavior exists in soil has yet to be verified. Considering vertical agar plates have been extensively and successfully used to study regulatory mechanisms of Arabidopsis roots, the simplified system will be useful to identify major genetic components operating in soil-growing roots.

We show that ARG is specifically caused by the deficiency of nitrate but not other nitrogen sources, such as NH_4_^+^ or Glu (Supplementary Figure S4). In fact, the addition of NH_4_^+^ or Glu under nitrate deficient condition even resulted in an enhanced ARG (Supplementary Figure S4) likely due to antagonistic roles of other nitrogen sources on endogenous nitrate signaling. Nitrate deficiency–induced ARG is unlikely to be resulted from reduced nitrate uptake since functional loss of *NRT1.2* or *NRT2.1* did not grow in coils as that of *NRT1.1*, as in *chl1-5* (Figure 5). Another *NRT1.1* mutant we used, *chl1-9*, is defective in nitrate uptake but not in nitrate sensing (Ho et al., 2009). Root growth of *chl1-9*, unlike that of *chl1-5*, responded to nitrate in the medium comparably to that of wild type (Figure 5), suggesting that NRT1.1 mediates nitrate suppression of ARG mainly by sensing environmental nitrate.

Although the growth behavior of roots is determined by a combination of gravitropism, thigmotropism, and circumnutation (Migliaccio and Piconese, 2001; Migliaccio et al., 2009, 2013), our results suggested that responses to gravity and mechanical stresses are not the major cause of the strong ARG induced by a nitrate deficiency (Supplementary Figures S2, S3) or a functional loss of *NRT1.1* (Supplementary Figure S10). In addition, changes of root system architecture (RSA) by N supply could not explain the results. Although the N deficiency or the functional loss of *NRT1.1* under nitrate-limiting conditions enhances the growth of lateral roots (Krouk et al., 2010; Gruber et al., 2013), they do not have much impact on the growth of primary roots (Supplementary Figure S1). Indeed, the growth of primary roots did not differ between 7N medium and 0N medium or between wild type and *chl1-5* on 7N medium (Supplementary Figure S8). However, the growth rate of primary roots does have an impact on the degree of ARG. The deficiency of potassium, sucrose, or phosphate reduced the root growth (Supplementary Figure S1) and caused a significantly reduced ARG as compared to that with full nutrients (Figure 1), suggesting that the growth rate of primary roots influences the degree of ARG.

Mutational studies in the past hinted at the critical role of MT dynamics in root circumnutating growth (Buschmann et al., 2004; Ishida et al., 2007; Bisgrove et al., 2008). Here we showed that nitrate deficiency–induced ARG accompanies skewing of cortical MT deposition (Figure 2). Instead of transverse arrays of cortical MT, nitrate deficiency induced tilting or skewing of MTs in root epidermal cells before roots substantially turned (Figure 2). The application of oryzalin or taxol suppressed the nitrate deficiency–induced root coiling (Supplementary Figure S5), further supporting the involvement of dynamic MT organization.

ARG induced by a nitrate deficiency or by a functional loss of *NRT1.1* is suppressed by NPA (Figures 3, 6) or a functional loss of *PIN2* or *AUX1* (Figures 4, 7), which compromised polar auxin transport (Boutte et al., 2007; Band et al., 2014). Earlier studies showed that NRT1.1 acts locally to modulate auxin level by transporting auxin away from lateral root tips (Krouk et al., 2011; Mounier et al., 2014). However, the distribution of DR5:GFP was comparable between wild type on 0N medium (Figure 3) and *chl1-5* on 7N medium (Figure 7), excluding the possibility that NRT1.1-mediated auxin transport participates in this process.

The dependency of root coiling on polar auxin transport is similar to that during gravitropism (Chen et al., 1998; Luschnig et al., 1998; Friml et al., 2002; Swarup et al., 2005; Abas et al., 2006). Upon gravitropic stimulation, AUX1 loads cells with auxin while PIN2 directs auxin into the elongation zone at the lower side of the roots, leading to its inhibition (Swarup et al., 2005; Abas et al., 2006). Unlike gravitropic curvature that is primarily driven by the differential expansion of root epidermal cells in the elongation zone (Swarup et al., 2005; Abas et al., 2006; Baster et al., 2013), both unequal cell proliferation and elongation contributed to nitrate deficiency–induced ARG (Figure 2). The difference may be due to different time frames. Gravitropic responses are usually observed during a short period when differential cell elongation can provide a rapid solution for asymmetry. By contrast, root coiling as examined here occurs at a much longer term, and therefore enhanced cell division (Figure 2) would be a supplementary approach to ensure ARG.

## Data Availability Statement

All datasets generated for this study are included in the article/Supplementary Material.

## Author Contributions

SL: conceptualization and supervision. SC and EL: investigation and methodology. YZ and SL: writing and funding acquisition.

## Conflict of Interest

The authors declare that the research was conducted in the absence of any commercial or financial relationships that could be construed as a potential conflict of interest.

## Footnotes

1 http://www.phytotechlab.com/

2 http://rsbweb.nih.gov/ij/

3 www.graphpad.com/scientific-software/prism
