# Supplementary material for: NRT1.1-Mediated Nitrate Suppression of Root Coiling Relies on PIN2- and AUX1-Mediated Auxin Transport
Source: Front Plant Sci. 2020 Jun 4;11:671. doi: 10.3389/fpls.2020.00671 (PMC7288464; doi:10.3389/fpls.2020.00671)
Supplement: Supplementary file 1 [file Data_Sheet_1.PDF]

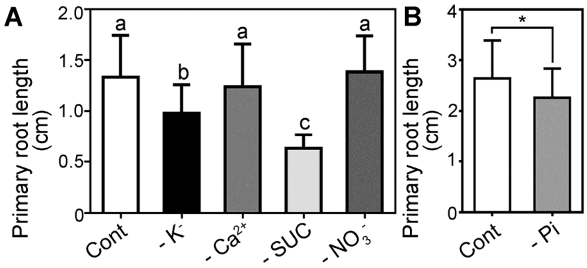

Figure S1. Primary root growth upon nutrient deficiency.

(A) Primary root length on medium deficient in in potassium (-K^+), calcium (-Ca^{2+}), sucrose (-Suc), or nitrate (-NO_3^-). (B) Primary root length of Arabidopsis growing on 1 mM phosphorus medium (Cont) or phosphorus-deficient medium (-Pi). Results shown in (A, B) are means ± standard error (SE). Three independent experiments involving 30 roots in each treatment were conducted. Different letters in (A) indicate significant difference in root length (One-Way ANOVA, Tukey's multiple comparisons test, $P<0.05$). Asterisk in (B) indicates significant difference (*t*-test, $P<0.05$).

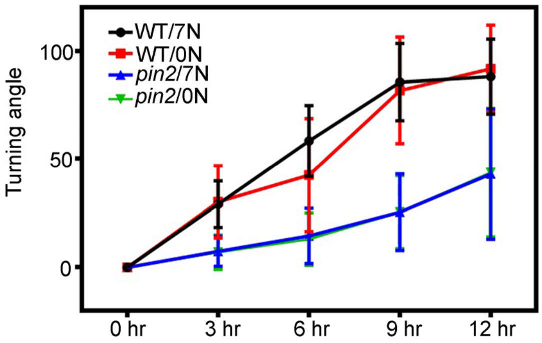

Figure S2. Nitrate deficiency does not substantially affect response to gravity.
Turning angle during gravitropic stimulation. The mutant *pin2* was used as a control for gravitropic stimulation. Results shown are means ± SE. Three independent experiments involving 30 roots in each treatment were conducted. Wild type showed significantly different gravitropic responses between 0N and 7N only at 6 hr after gravitropic stimulation (*t*-test, P<0.05).

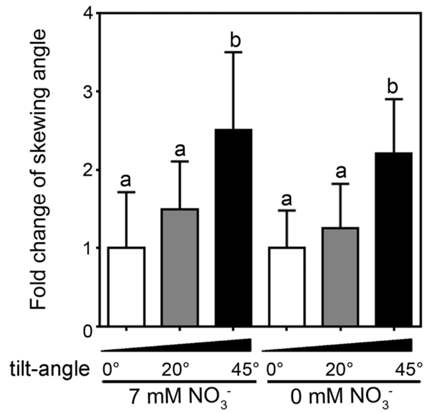

Figure S3. Nitrate deficiency does not substantially affect response to mechanical stress.

Fold change of wild-type skewing angles upon different tilt-angles on 7 mM NO_3^- or 0 mM NO_3^- medium with 1% agar. Results shown are means ± SE. Three independent experiments involving 30 wild-type roots in each treatment were conducted. Means with different letters are significantly different from each other (One-way ANOVA, Tukey's multiple comparisons test, $P<0.05$).

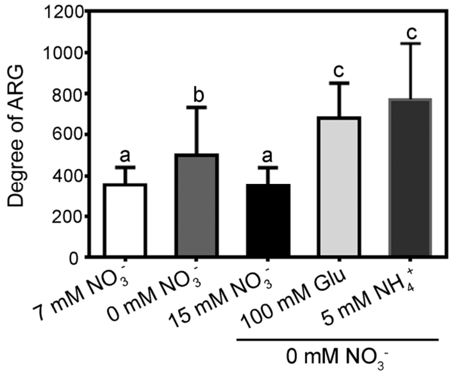

Figure S4. Other nitrogen sources could not replace nitrate in suppressing ARG. Degree of ARG upon different nitrogen sources. Results shown are means ± SE. Three independent experiments involving 30 roots in each treatment were conducted. Means with different letters are significantly different at ARG degrees (One-way ANOVA, Tukey's multiple comparisons test, $P<0.05$).

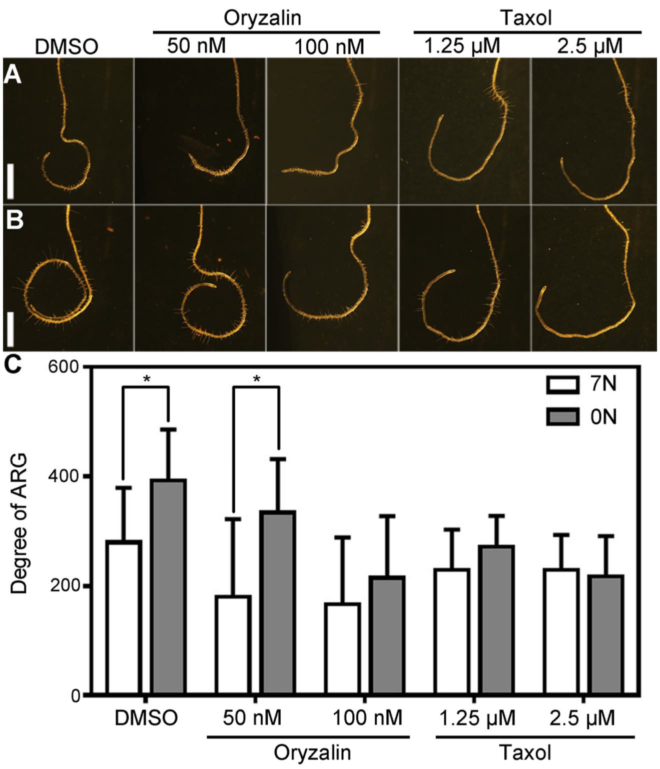

Figure S5. Pharmacological interference of MT organization impaired nitrate deficiency-induced root coiling.

(A-B) Representative roots from wild type on 7N medium (A) or 0N medium (B) upon treatment with DMSO, with different concentrations of Oryzalin or Taxol. (C) Degree of ARG on 7N (open bars) or 0N medium (filled bars). Results are means ± SE. Three independent experiments involving 30 roots in each treatment were conducted. Asterisks indicate significant difference of the treatment between 7N and 0N medium (*t*-test, $P<0.05$). Bars = 1 mm.

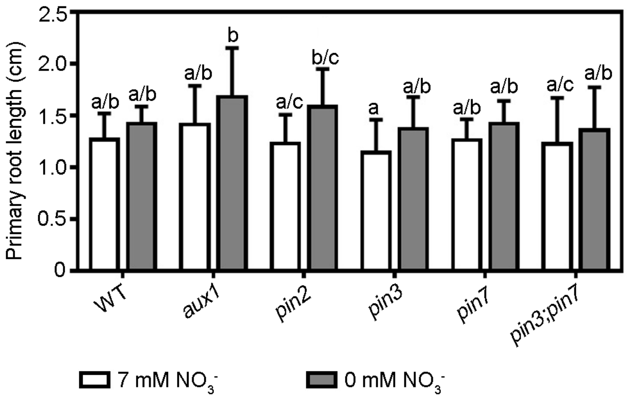

Figure S6. Primary root growth of auxin-transporter mutants upon nitrate deficiency. Primary root length of wild type, *pin2*, *aux1*, *pin3*, *pin7*, and *pin3;pin7* on 7N (open bars) or 0N medium (filled bars). Results shown are means ± SE. Three independent experiments involving 30 roots in each treatment were conducted. Means with different letters indicate significant different groups (One-Way ANOVA, Tukey's multiple comparisons test, $P<0.05$).

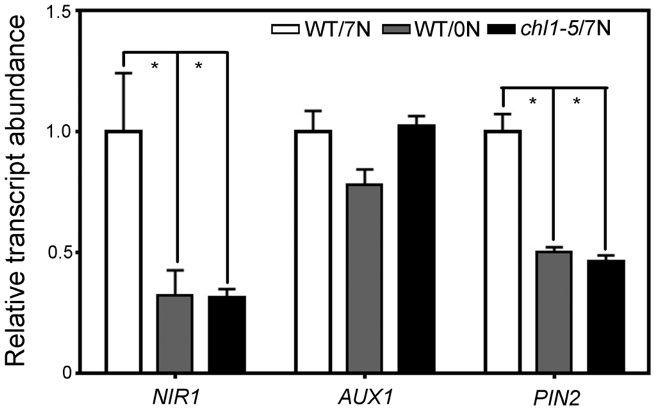

Figure S7. Expression of *PIN2* and *AUX1* upon nitrate deficiency or in *chl1-5*. Quantitative real-time PCRs showing the relative transcript abundance of *NIR1, AUX1* and *PIN2* in different genetic backgrounds. Results shown are means ± SE. Three biological replicates were analyzed. Asterisks indicate significant difference (*t*-test, P<0.05).

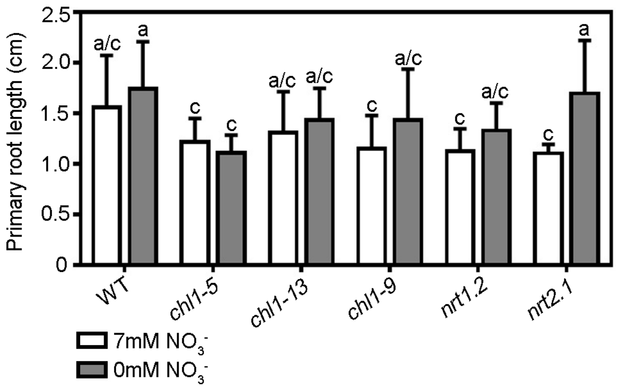

Figure S8. Primary root growth of nitrate transporter mutants upon nitrate deficiency.

Primary root length of wild type, *chl1-5*, *chl1-13*, *chl1-9*, *nrt1.2*, or *nrt2.1* on 7N (open bars) or 0N medium (filled bars). Results shown are means ± SE. Three independent experiments involving 30 roots in each treatment were conducted. Means with different letters indicate significant different groups (One-Way ANOVA, Tukey's multiple comparisons test, P<0.05).

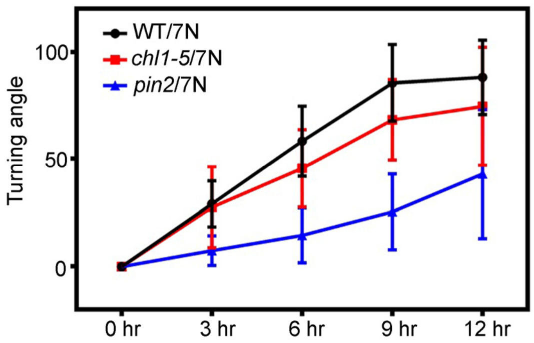

Figure S9. Functional loss of *NRT1.1* does not substantially affect gravitropic response.

Turning angle of wild type, *chl1-5*, or *pin2* during gravitropic stimulation. Results shown are means ± SE. Three independent experiments involving 30 roots in each treatment were conducted. *chl1-5* showed significantly different gravitropic response from wild type only at 6 hr after gravitropic stimulation (*t*-test, P<0.05).

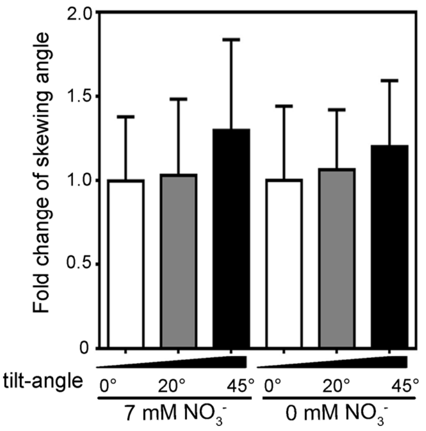

Figure S10. Functional loss of *NRT1.1* does not substantially affect responses to mechanical stress.

Fold change of *chl1-5* skewing angles upon different tilt-angles on 7 mM NO_3^- or 0 mM NO_3^- medium with 1% agar. Results shown are means ± SE. Three independent experiments involving 30 *chl1-5* roots in each treatment were conducted. All treatments were not significantly different from each other (One-way ANOVA, Tukey's multiple comparisons test, P>0.05).

Table S1. Oligos used in this study.

**Application**		**5'-3'sequences**
qPCRs	*ACTIN*	CGTGACCTTACTGATTAC
		TTCTCCTTGATGTCTCTT
	GAPDH	TGAAATCAAAAAGCTATCAAGG
		CATCATCCTCGGTGTATCCAA
	NIR1	CCGGTAGCCAGTTCTGCG
		CCTATTCGTCCCCCGACGT
	AUX1	TTGGTTCAGCTGCGCATCTA
		GCAGTCCAGCTTCCTAGTAA
	PIN2	CCGTGGGGCTAAGCTTCTCATCT
		AGCTTTCCGTCGTCTCCTATCTCC
